# Supplementary material for: Mutations in Parkinsonism-linked endocytic proteins synaptojanin1 and auxilin have synergistic effects on dopaminergic axonal pathology
Source: NPJ Parkinsons Dis. 2023 Feb 15;9:26. doi: 10.1038/s41531-023-00465-5 (PMC9932162; doi:10.1038/s41531-023-00465-5)
Supplement: Supplementary file 1 — Supplementary materials [file 41531_2023_465_MOESM1_ESM.pdf]

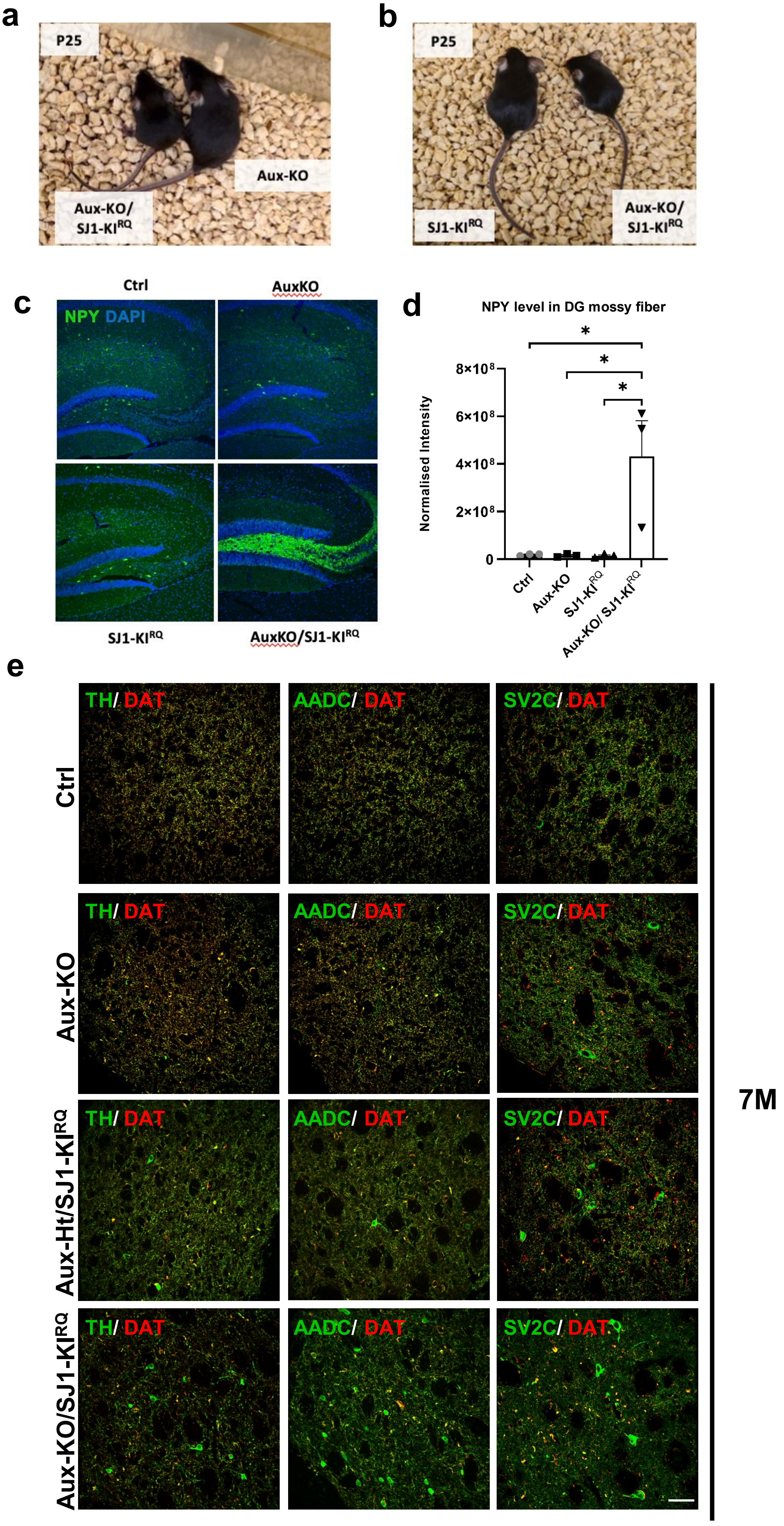


**Supplementary Fig 1**

**Supplementary Figure 1. Aux-KO/SJ1-KI$^{RQ}$ mice are smaller in size and has more severe DAergic phenotype at 7-month-old.**

(a-b) Image of the size of P25 Aux-KO/SJ1-KI$^{RQ}$ mice comparing with its littermate single mutants; (a) Aux-KO and (b) SJ1-KI$^{RQ}$.

(c) Immunostaining of NPY shows elevated expression of NPY in the dentate gyrus of Aux-KO/SJ1-KI$^{RQ}$ mice.

(d) Quantification of fluorescence intensity of NPY staining shown in (c). Data are represented as mean ± SEM (* $p<0.05$ by one-way ANOVA with post-hoc Tukey's test). N = 3 mice for each genotype.

(e) Representative images for double immunofluorescence of TH/DAT, AADC/DAT and SV2C/DAT for all 4 genotypes at 7-month-old. The only Aux-KO/SJ1-KI$^{RQ}$ that managed to survive till this age showed increased number of clusters, THINs and ChINs compared to the other 3 genotypes. Scale bar: 50 µm.

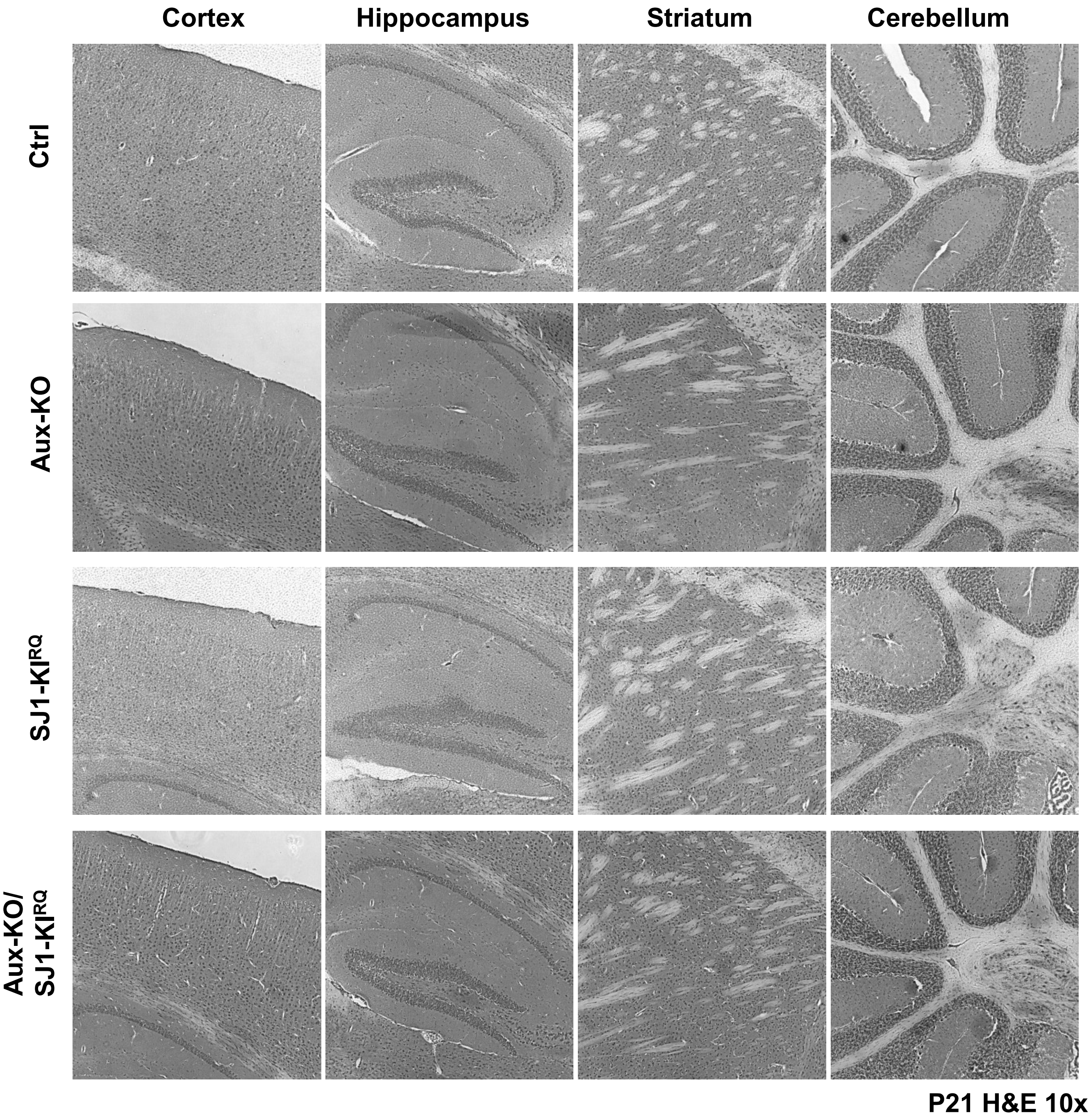


**Supplementary Fig 2**

**Supplementary Figure 2. Aux-KO/SJ1-KI$^{RQ}$ mice have normal brain development and architecture.**

H&E staining reveals absence of gross structural abnormality in various regions (cortex, hippocampus, striatum and cerebellum) of 1-month-old Aux-KO, SJ1-KI$^{RQ}$ and Aux-KO/SJ1-KI$^{RQ}$ mice brain.

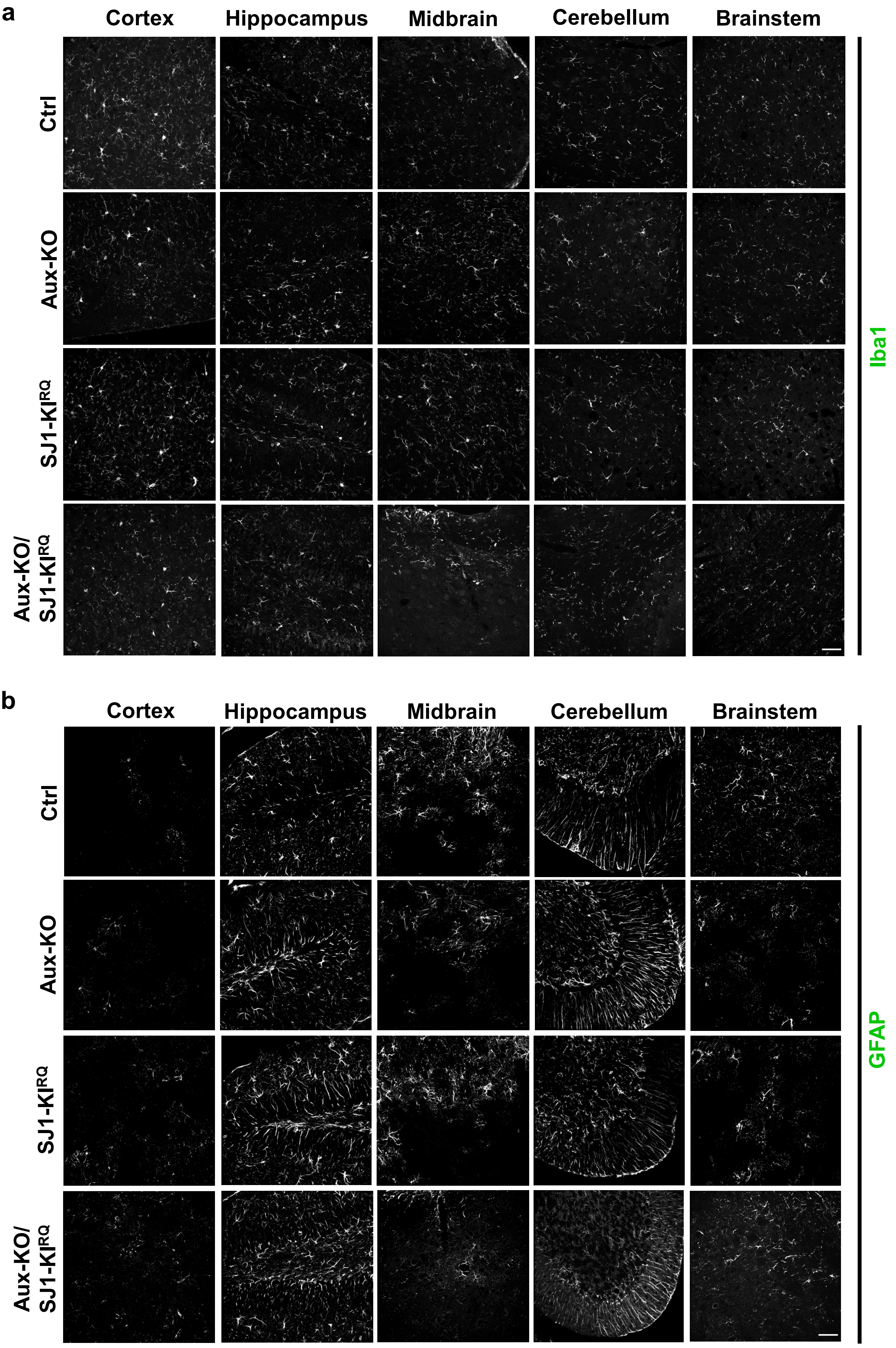


**Supplementary Fig 3**

**Supplementary Figure 3. Absence of gliosis in Aux-KO/SJ1-KI$^{RQ}$ brain.**

(a and b) No obvious difference in immunostaining intensity of (a) Iba1-microglia marker and (b) GFAP-astrocytes marker in different regions of 1-month-old Ctrl, Aux-KO, SJ1-KI$^{RQ}$ and Aux-KO/SJ1-KI$^{RQ}$ mice brain. Scale bar: 50 μm.

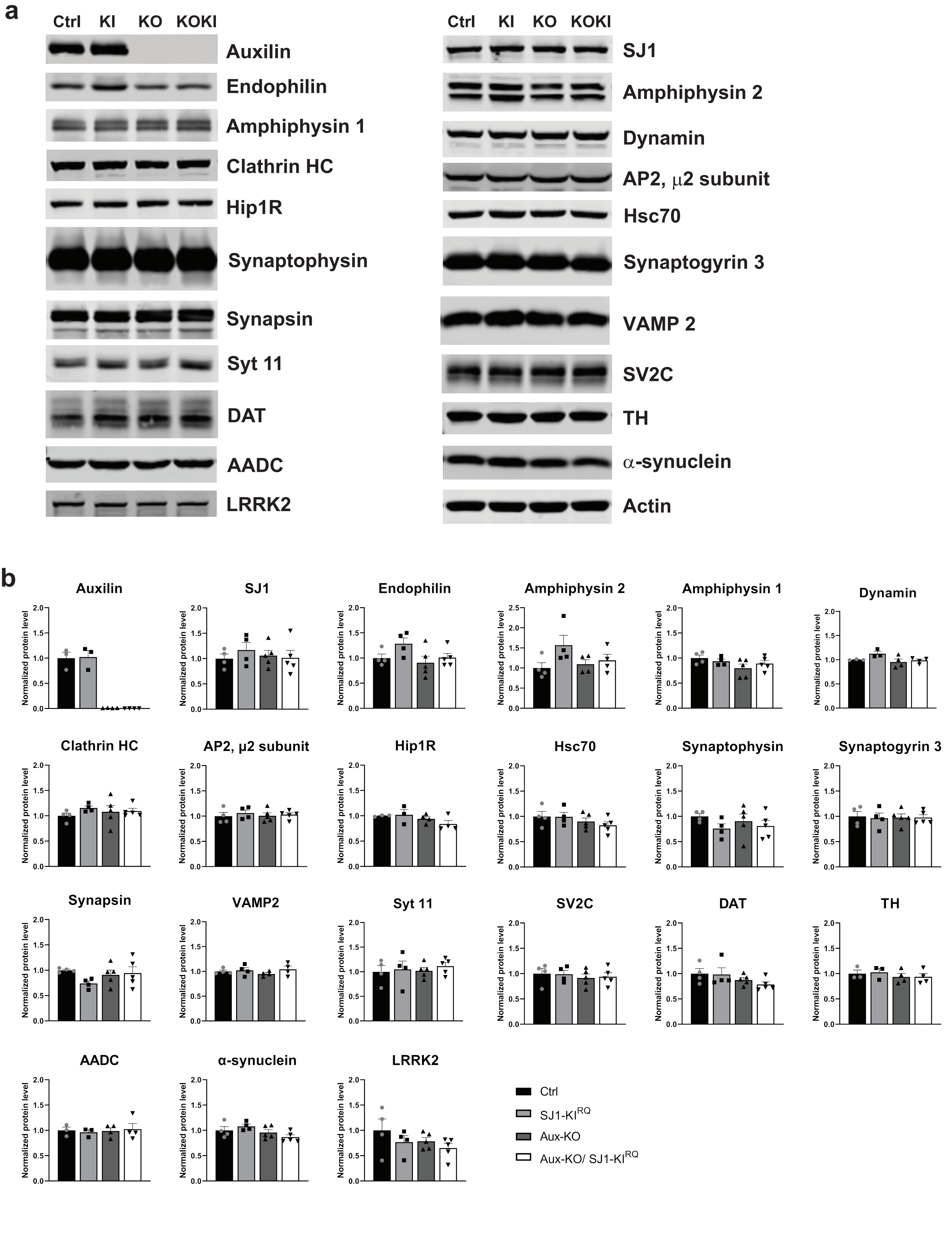


Supplementary Fig 4

**Supplementary Figure 4. Western blot analysis of endocytic proteins, synaptic proteins, DAergic markers and PD-related proteins in mouse brains.**

(a) Representative blots for various proteins involved in PD and synaptic endocytosis from 1-month-old control, SJ1-KI$^{RQ}$, Aux-KO and Aux-KO/SJ1-KI$^{RQ}$ whole brain homogenates.

(b) Quantification of expression levels of the proteins shown in (a). Protein levels were normalized to the level of Actin. Data are represented as mean ± SEM (one-way ANOVA with post-hoc Tukey's test). N = 3-4 mice for each genotype.

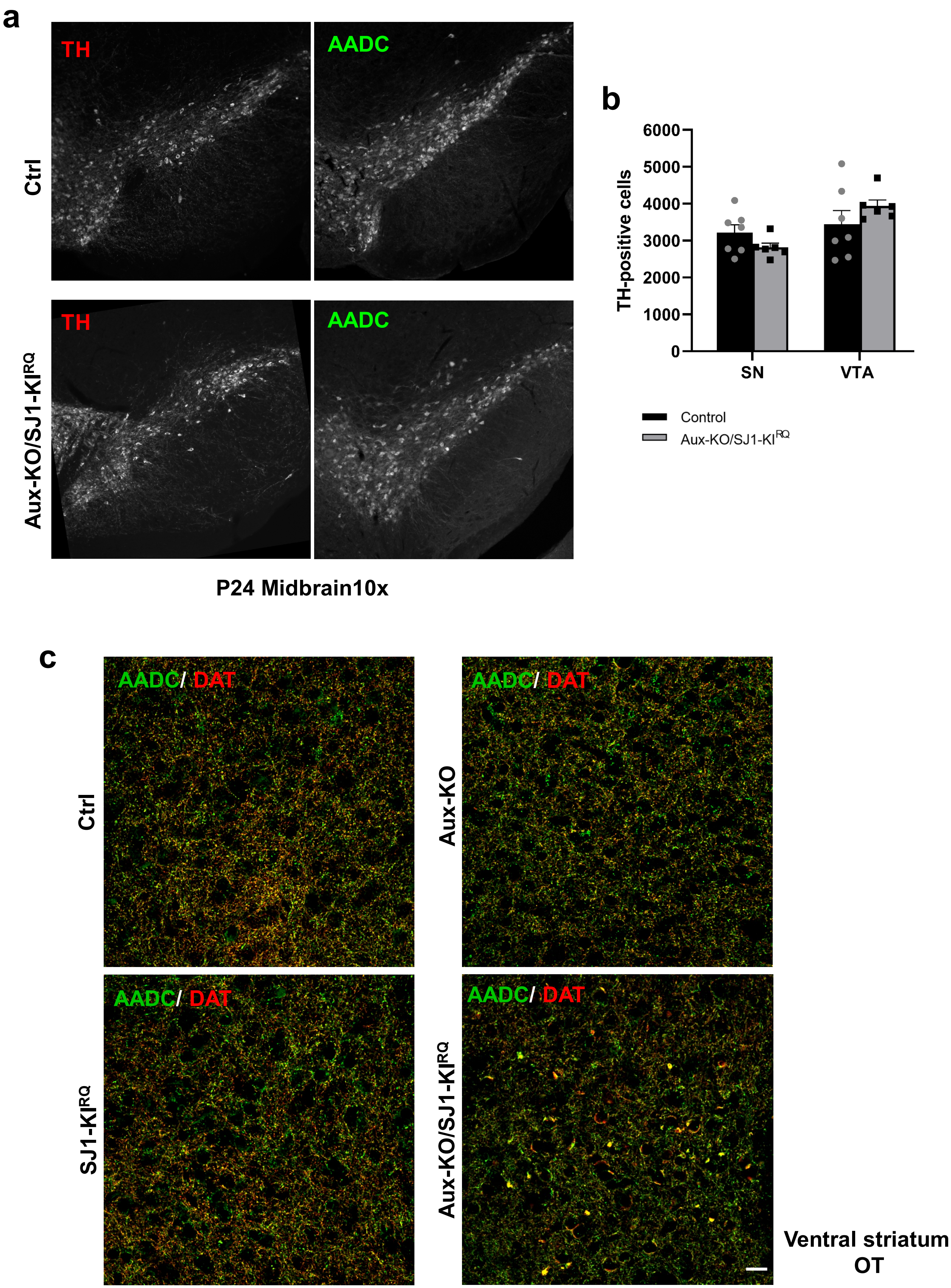


Supplementary Fig 5

**Supplementary Figure 5. Presence of dystrophic changes only in the axon terminals of DAergic neurons but not the cell bodies of Aux-KO/SJ1-KI$^{RQ}$ mice.**

(a) Double staining of anti-TH and anti-AADC showed normal morphology of DAergic neuron cell bodies and dendrites in the midbrain.

(b) Stereological analysis of TH-positive cell bodies in the SN and VTA region of control and Aux-KO/SJ1-KI$^{RQ}$ mice at 1 month old. The estimated cell numbers in one hemisphere is shown. Data are represented as mean ± SEM (Student's unpaired t-test). n=7 mice for control whereas n=6 mice for Aux-KO/SJ1-KI$^{RQ}$.

(c) AADC (green) and DAT (red) immunostaining reveals the presence of AADC/DAT-positive aggregates in the olfactory tubercle (OT, part of ventral striatum) of Aux-KO/SJ1-KI$^{RQ}$ mice. Scale bar: 20 μm.

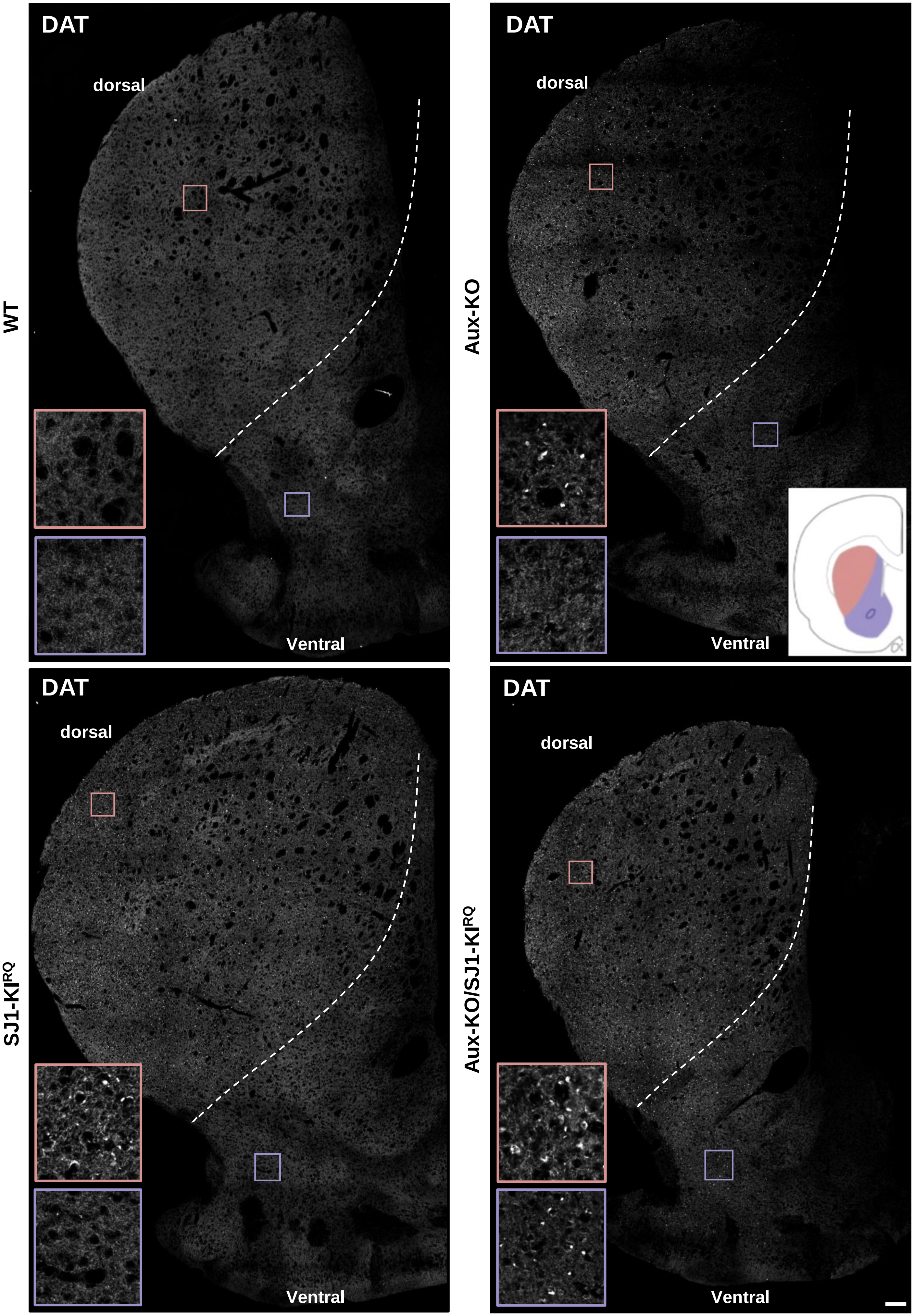


**Supplementary Fig 6**

**Supplementary figure 6. Overview of cluster distribution in the striatum of Aux-KO, SJ1-KI$^{RQ}$ and Aux-KO/SJ1-KI$^{RQ}$ mice.**

Tiling of a single coronal striatum section immunostained for DAT. White dotted line separates the dorsal and ventral striatum of the mice. Insets on the bottom left corner of the image showed high magnification images of the clusters for dorsal (pink, top) and ventral (purple, bottom) striatum for each genotype. Note the absence of clusters in the ventral striatum in all mice except the Aux-KO/SJ1-KI$^{RQ}$ mice. Bottom right inset of the Aux-KO tiling image depicts the dorsal (pink) and ventral (purple) mouse striatum. Scale bar: 100 µm.

a

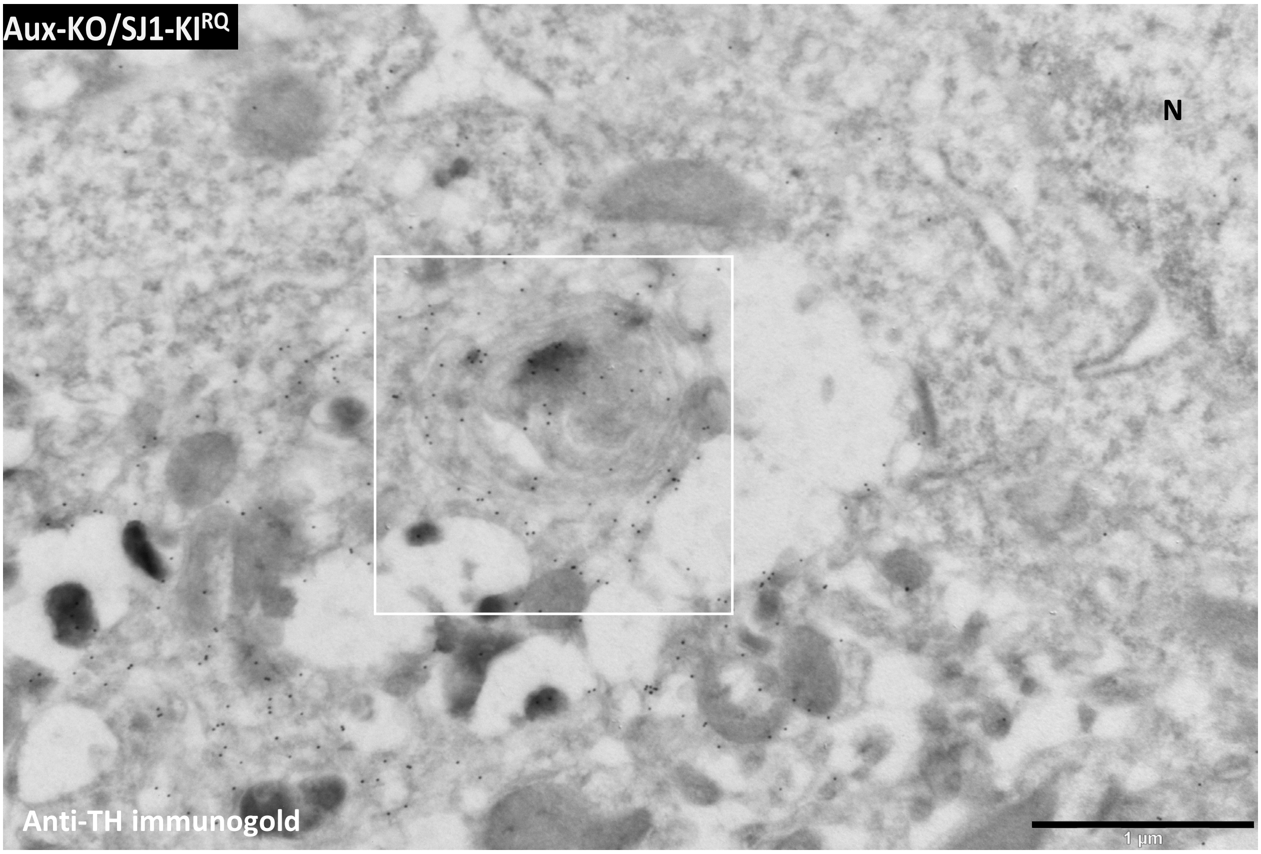


b

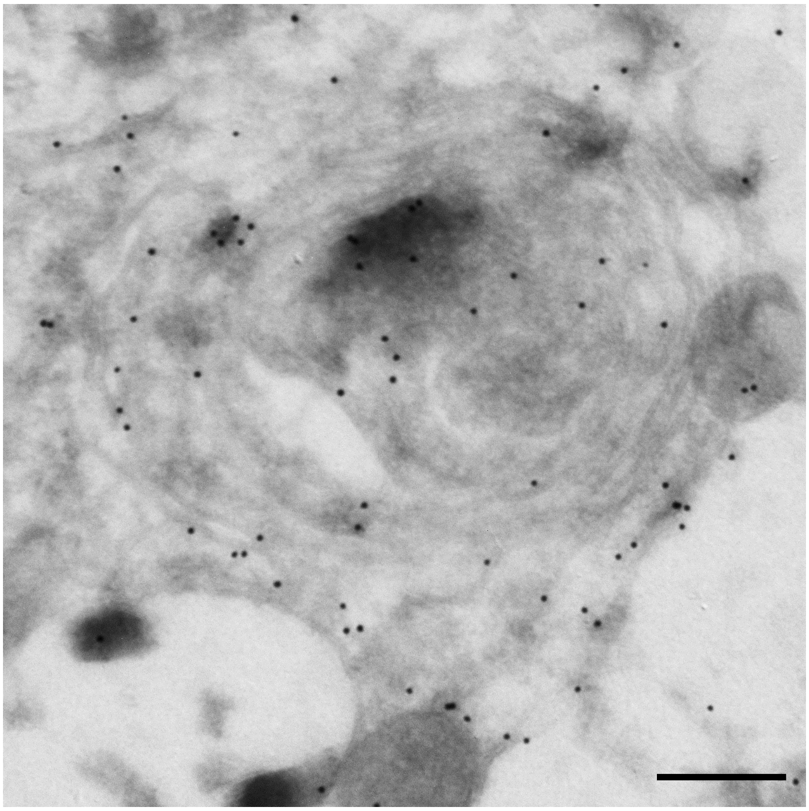

**Supplementary Fig 7**

**Supplementary figure 7. EM immunogold labelling of onion-like membrane structure in dorsal striatum of Aux-KO/SJ1-KI$^{RQ}$ mice.**

(a) Anti-TH immunogold labelling (10 nm gold particles) of an ultrathin frozen sections of Aux-KO/SJ1-KI$^{RQ}$ dorsal striatum. The multilayered membrane structures are positive for TH immunoreactivity (boxed in white box). Scale bar: 1 μm

(b) The onion-like membrane structures boxed in white in (a) is enlarged and shown here. Scale bar: 250 nm

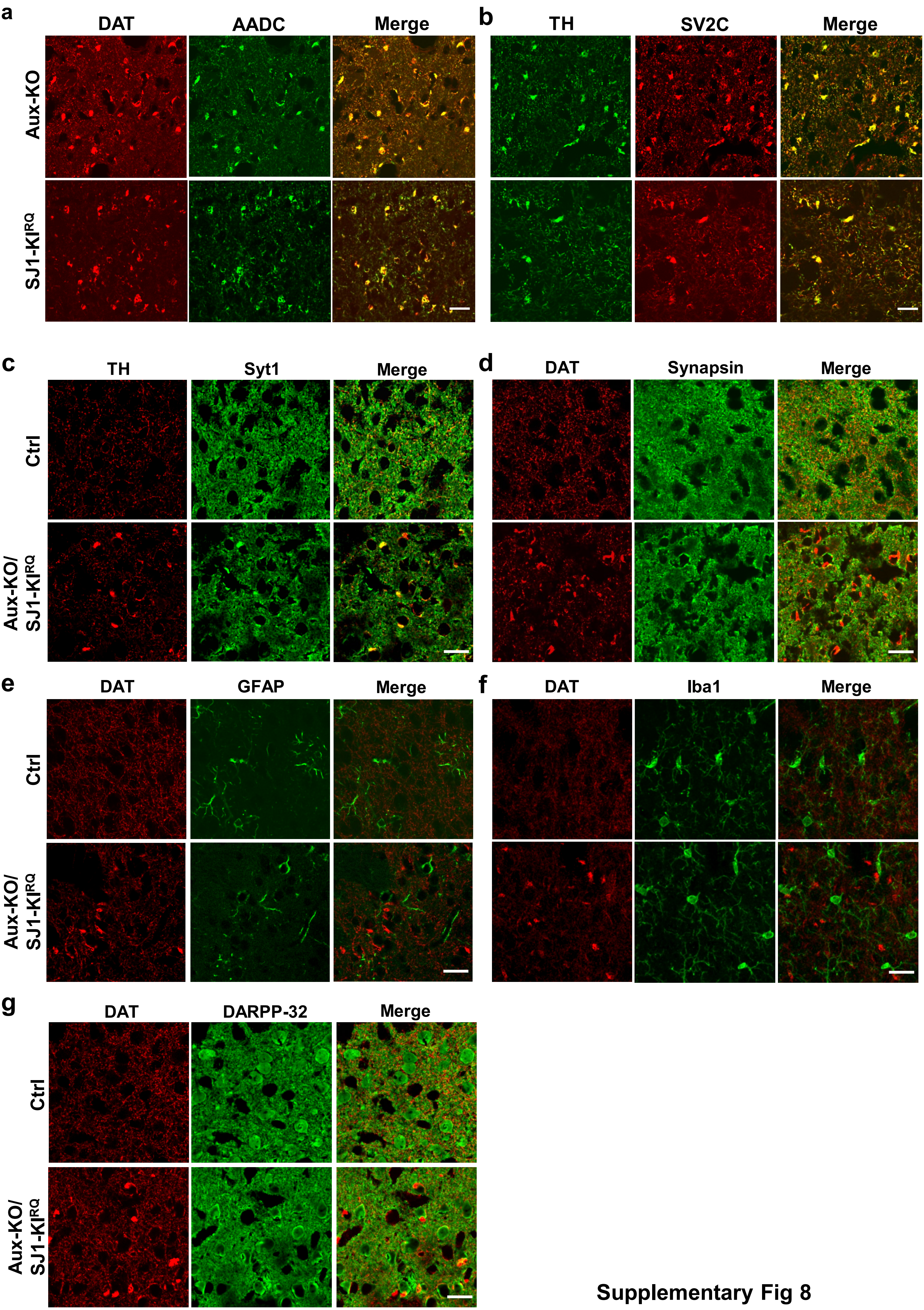


**Supplementary Fig 8**

**Supplementary figure 8. Various other markers examined with TH/DAT-positive clusters.**

(a-b) Representative images for immunoreactivity of anti-DAT or TH with (a) anti-AADC and (b) anti-SV2C in Aux-KO and SJ1-KI$^{RQ}$ mice. Scale bar: 20 μm.

(c) Immunostaining of DAT with Syt1 showed partial colocalization in Aux-KO/SJ1-KI$^{RQ}$ double mutant striatum. Scale bar: 20 μm.

(d-g) Double immunofluorescence of synapsin, GFAP, Iba1 and Darpp32 with TH/DAT showed no colocalization in the striatum of Aux-KO/SJ1-KI$^{RQ}$ mice. Scale bar: 20 μm.

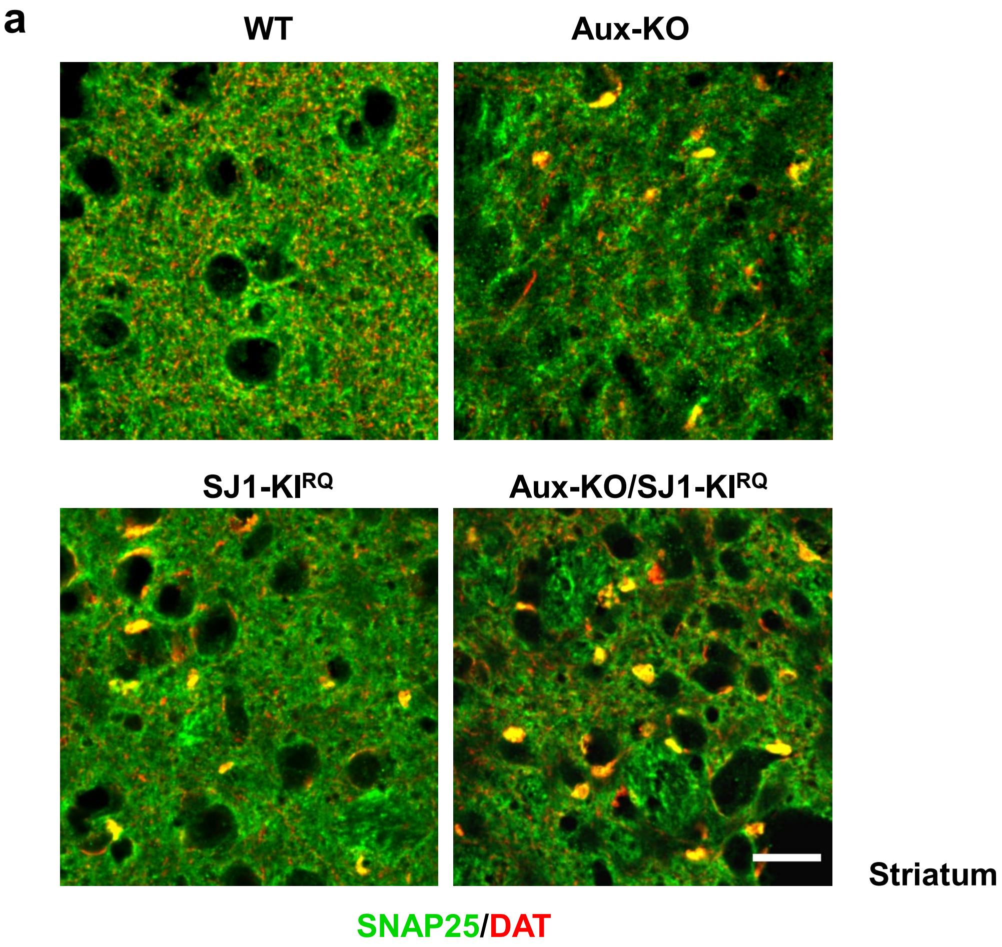


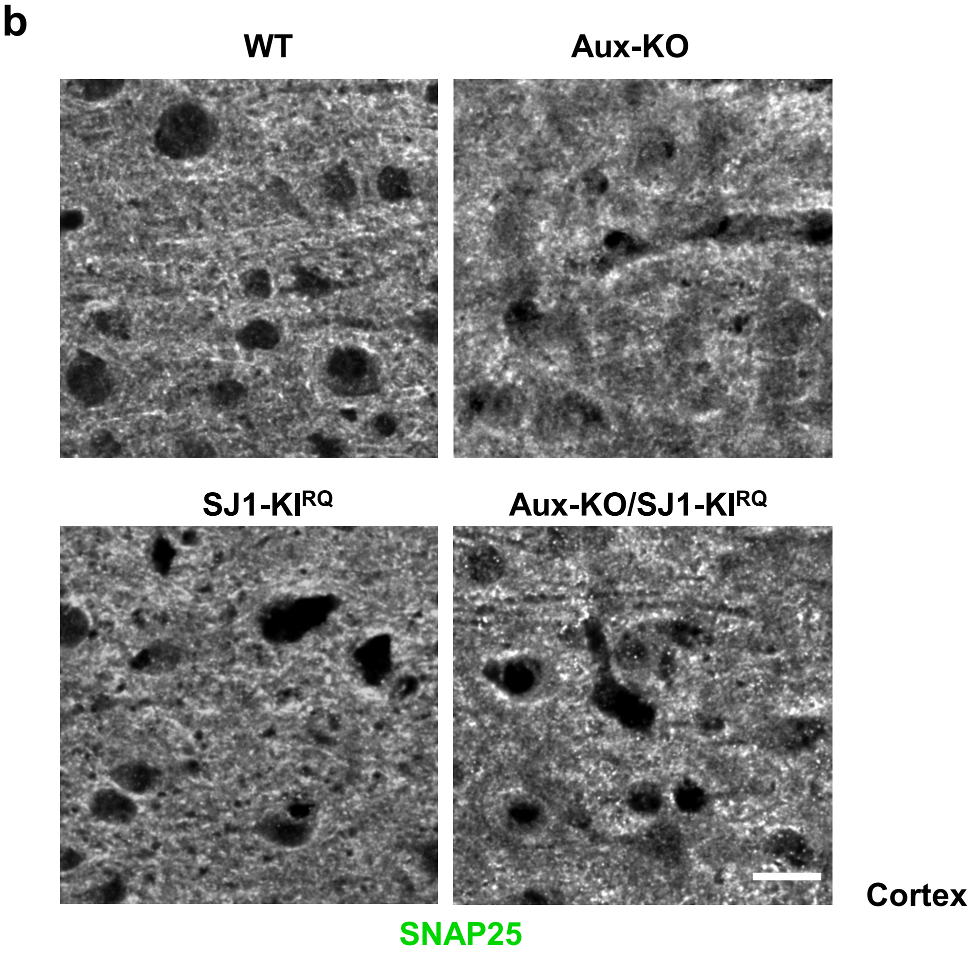


Supplementary Fig 9

**Supplementary figure 9. Immunofluorescence analysis of SNAP25 in different brain regions.**

(a) Immunostaining of SNAP25 (green) showed partial colocalization with DAT-positve clusters (red) in the striatum of Aux-KO, SJ1-KI$^{RQ}$ and Aux-KO/SJ1-KI$^{RQ}$ mice. Scale bar: 20 μm.

(b) Immunoreactivity of anti-SNAP25 in the cortex of WT, Aux-KO, SJ1-KI$^{RQ}$ and Aux-KO/SJ1-KI$^{RQ}$ mice. No clusters of SNAP25 were observed in the cortex. Scale bar: 20 μm.

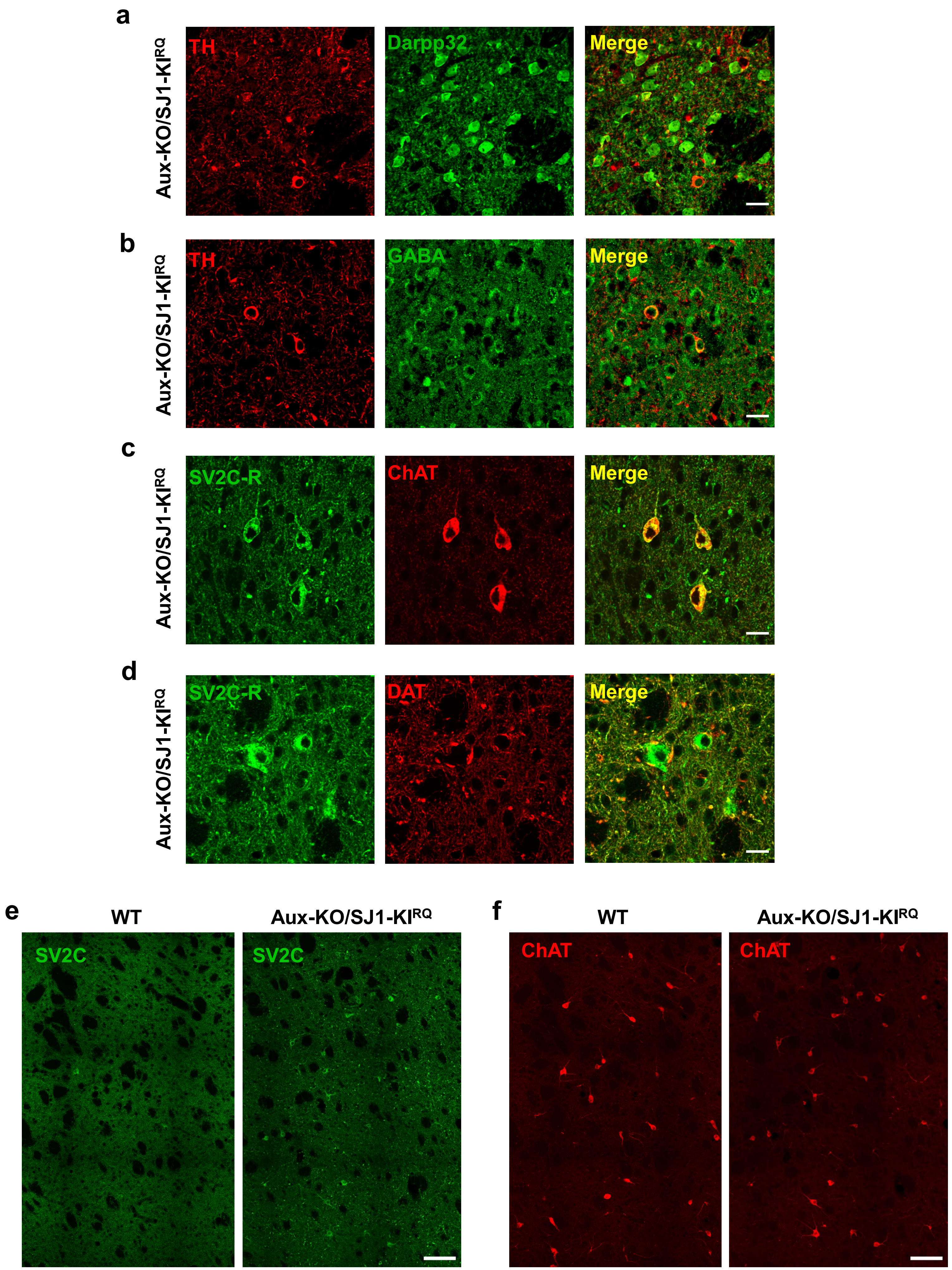


**Supplementary Fig 10**

**Supplementary figure 10. Properties of striatal THINs and ChINs in the striatum of Aux-KO/SJ1-KI$^{RQ}$ mice.**

(a) Immunoreactivity of anti-TH with anti-Darpp32 showed that THINs is a distinct group of neurons from Darpp32-positive MSNs. Scale bar: 20 μm.

(b) Double staining of anti-TH and anti-GABA showed that THINs are GABAergic striatal interneurons. Scale bar: 20 μm.

(c-d) Immunostaining of SV2C using a different anti-SV2C antibody (host species: Rabbit) labelled the same structures: (c) ChAT-positive ChINs and (d) SV2C/DAT-positive clusters in Aux-KO/SJ1-KI$^{RQ}$. Scale bar: 20 μm.

(e-f) A large microscope field of view showed the distribution of (e) SV2C-positive and (f) ChAT-positive interneurons in the striatum of WT and Aux-KO/SJ1-KI$^{RQ}$ mice. Scale bar: 100 μm.

**Supplementary Video Legends**

**Supplementary Video 1 Tonic-clonic epileptic seizures in a 5-month-old Aux-KO mouse.**

**Supplementary Video 2 Balance beam test in 2-month-old (a) Ctrl and (b) Aux-KO mice.**

**Supplementary Video 3 Balance beam test in 4-month-old (a) Ctrl, (b) Aux-KO/SJ1-WT, (c) Aux-Ht/SJ1-KI$^{RQ}$ and (d) Aux-KO/SJ1-KI$^{RQ}$ mice.** This 4-month-old Aux-KO/SJ1-KI$^{RQ}$ mouse is the same mouse that managed to survive till 7-month-old.
