# Supplementary figures and images for: Mutations in Parkinsonism-linked endocytic proteins synaptojanin1 and auxilin have synergistic effects on dopaminergic axonal pathology
Source: NPJ Parkinsons Dis. 2023 Feb 15;9:26. doi: 10.1038/s41531-023-00465-5 (PMC9932162; doi:10.1038/s41531-023-00465-5)

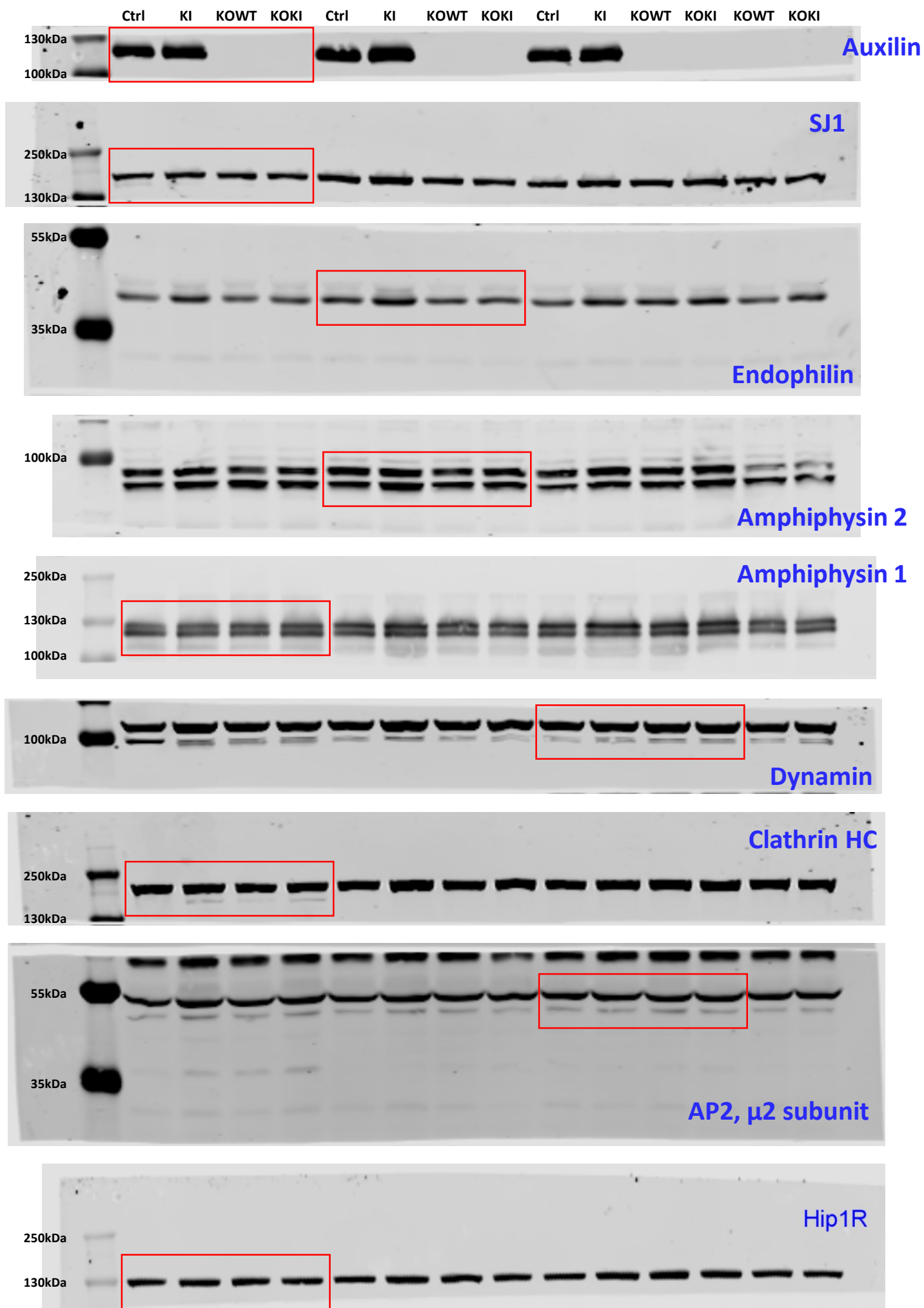

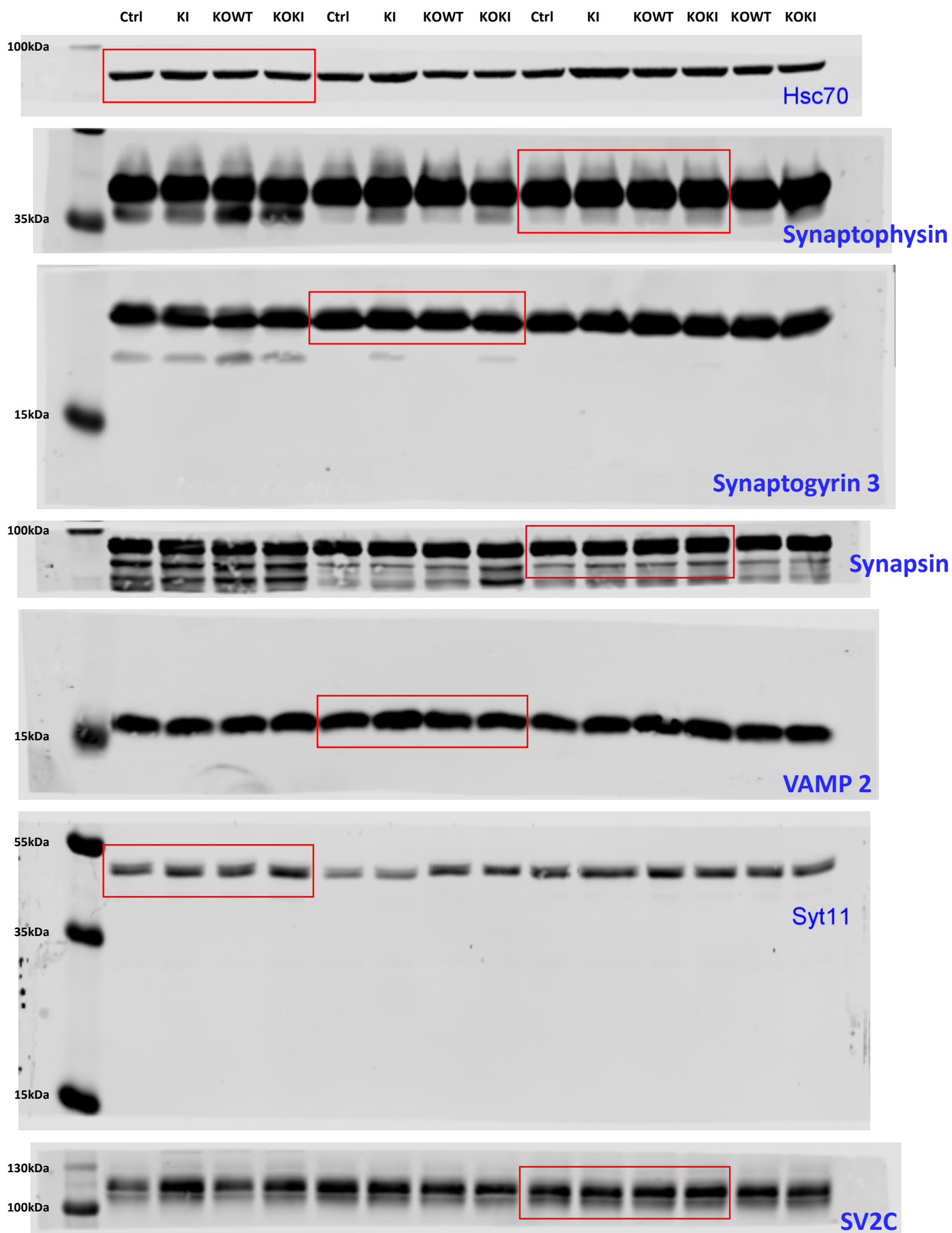

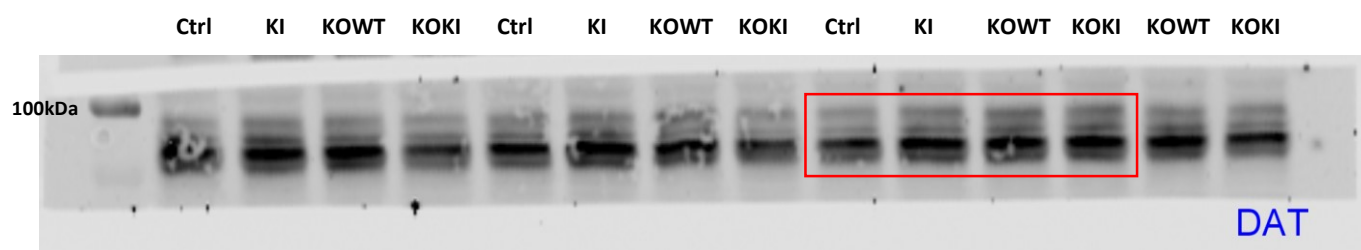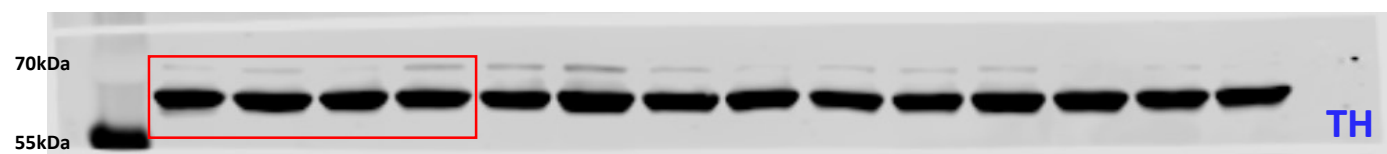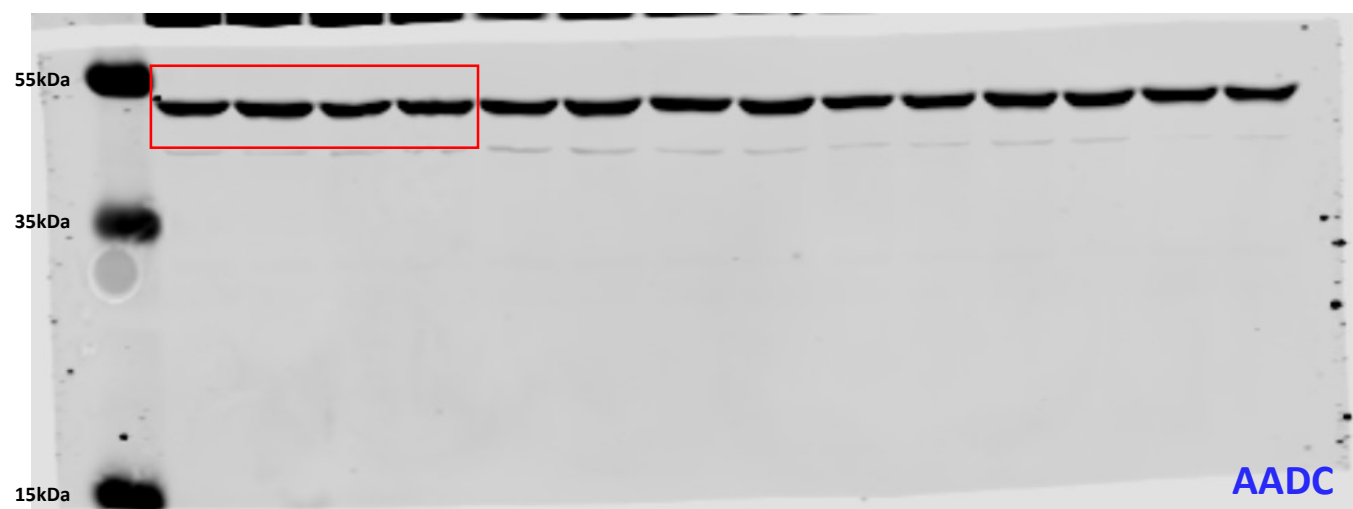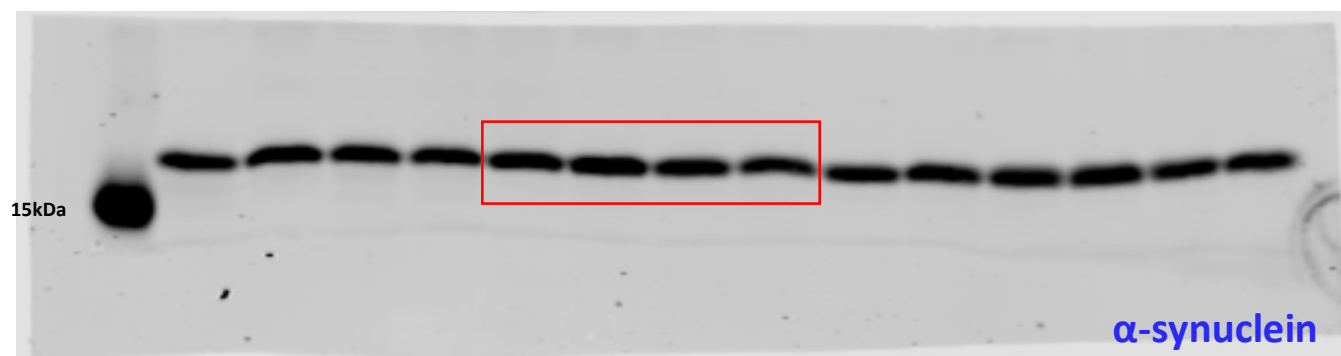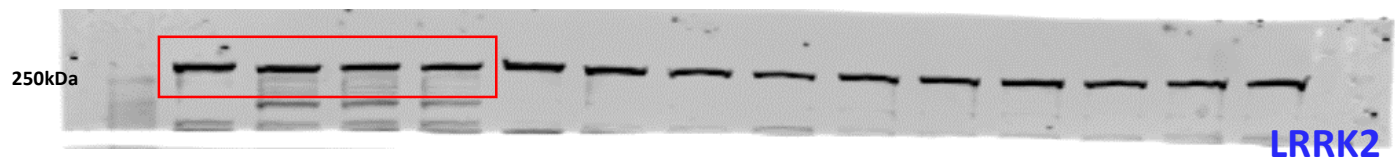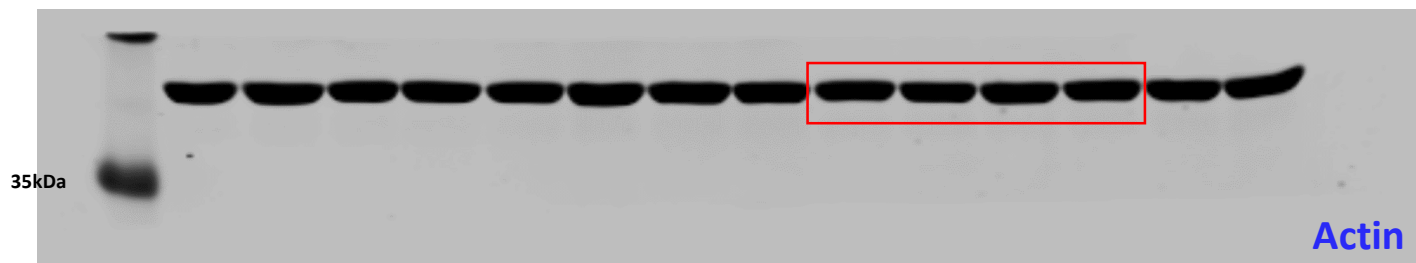

Supplement: Supplementary file 2 — uncropped blots [file 41531_2023_465_MOESM2_ESM.pdf]
