# Supplementary material for: Mutations in Parkinsonism-linked endocytic proteins synaptojanin1 and auxilin have synergistic effects on dopaminergic axonal pathology
Source: NPJ Parkinsons Dis. 2023 Feb 15;9:26. doi: 10.1038/s41531-023-00465-5 (PMC9932162; doi:10.1038/s41531-023-00465-5)
Supplement: Supplementary file 3 — Reporting Summary [file 41531_2023_465_MOESM3_ESM.pdf]

Corresponding author(s): Mian Cao

Last updated by author(s): 2023/01/11

Reporting Summary

Nature Portfolio wishes to improve the reproducibility of the work that we publish. This form provides structure for consistency and transparency in reporting. For further information on Nature Portfolio policies, see our Editorial Policies and the Editorial Policy Checklist.

Statistics

For all statistical analyses, confirm that the following items are present in the figure legend, table legend, main text, or Methods section.

n/a	Confirmed	
☐	☒	The exact sample size (*n*) for each experimental group/condition, given as a discrete number and unit of measurement
☐	☒	A statement on whether measurements were taken from distinct samples or whether the same sample was measured repeatedly
☐	☒	The statistical test(s) used AND whether they are one- or two-sided<br>*Only common tests should be described solely by name; describe more complex techniques in the Methods section.*
☒	☐	A description of all covariates tested
☒	☐	A description of any assumptions or corrections, such as tests of normality and adjustment for multiple comparisons
☐	☒	A full description of the statistical parameters including central tendency (e.g. means) or other basic estimates (e.g. regression coefficient) AND variation (e.g. standard deviation) or associated estimates of uncertainty (e.g. confidence intervals)
☒	☐	For null hypothesis testing, the test statistic (e.g. *F*, *t*, *r*) with confidence intervals, effect sizes, degrees of freedom and *P* value noted<br>*Give P values as exact values whenever suitable.*
☒	☐	For Bayesian analysis, information on the choice of priors and Markov chain Monte Carlo settings
☒	☐	For hierarchical and complex designs, identification of the appropriate level for tests and full reporting of outcomes
☒	☐	Estimates of effect sizes (e.g. Cohen's *d*, Pearson's *r*), indicating how they were calculated

Our web collection on statistics for biologists contains articles on many of the points above.

Software and code

Policy information about availability of computer code

Data collection	LI-COR Image Studio software (RRID: SCR_015795), Stereo Investigator software (RRID: SCR_002526), MetaMorph Microscopy Automation and Image Analysis Software (RRID: SCR_002368), ChromeleonTM 7.2 Chromatography Data System (RRID: SCR_016874), Thermo Scientific Velox software, iTEM Olympus software
Data analysis	Image Studio Lite software (Ver 5.2, RRID: SCR_013715), Fiji software (Version ImageJ 1.52p/Java 1.8.0_172 (64-bit), RRID: SCR_002285), and GraphPad Prism (Ver 9.3.1, RRID: SCR_002798)

For manuscripts utilizing custom algorithms or software that are central to the research but not yet described in published literature, software must be made available to editors and reviewers. We strongly encourage code deposition in a community repository (e.g. GitHub). See the Nature Portfolio guidelines for submitting code & software for further information.

Data

Policy information about availability of data

All manuscripts must include a data availability statement. This statement should provide the following information, where applicable:

- Accession codes, unique identifiers, or web links for publicly available datasets
- A description of any restrictions on data availability
- For clinical datasets or third party data, please ensure that the statement adheres to our policy

The datasets generated and/or analyzed in this study are available from the corresponding author Mian Cao on request.

Human research participants

Policy information about studies involving human research participants and Sex and Gender in Research.

Reporting on sex and gender	N/A
Population characteristics	N/A
Recruitment	N/A
Ethics oversight	N/A

Note that full information on the approval of the study protocol must also be provided in the manuscript.

Field-specific reporting

Please select the one below that is the best fit for your research. If you are not sure, read the appropriate sections before making your selection.

☒ Life sciences ☐ Behavioural & social sciences ☐ Ecological, evolutionary & environmental sciences

For a reference copy of the document with all sections, see nature.com/documents/nr-reporting-summary-flat.pdf

Life sciences study design

All studies must disclose on these points even when the disclosure is negative.

Sample size	Sample sizes were stated in the legend of the respective figures and supplementary figures. We did not use any statistical methods to predetermine sample sizes. We repeated all experiments with at least 3 individual mice (biological replicates) to meet the requirements for statistical analysis.
Data exclusions	In the stereological analysis of TH-positive DAergic neurons, 1 sample from the Aux-KO/SJ1-KI group was excluded. This is because its coefficient of error (Gunderson, m=1) exceeded the threshold of 0.1
Replication	All experiments were replicated for at least three times. All attempts at replications were successful.
Randomization	Mice were placed into their respective group based on their genotypes.
Blinding	Blinding was not possible as mice were genotyped before collection to ensure the right mice were collected for experiments.

Reporting for specific materials, systems and methods

We require information from authors about some types of materials, experimental systems and methods used in many studies. Here, indicate whether each material, system or method listed is relevant to your study. If you are not sure if a list item applies to your research, read the appropriate section before selecting a response.

Materials & experimental systems

n/a	Involved in the study	
☐	☒	Antibodies
☐	☒	Eukaryotic cell lines
☒	☐	Palaeontology and archaeology
☐	☒	Animals and other organisms
☒	☐	Clinical data
☒	☐	Dual use research of concern

Methods

n/a	Involved in the study	
☒	☐	ChIP-seq
☒	☐	Flow cytometry
☒	☐	MRI-based neuroimaging

Antibodies

Antibodies used	The following primary antibodies were obtained from these sources respectively:<br>1) Dr Pietro De Camilli's lab at Yale University: rabbit anti-SJ1 (Ock serum), rabbit anti-Auxilin (BABE serum), mouse anti-Amphiphysin 1 (#3), mouse anti-Clathrin Heavy Chain (TD1), rabbit anti-pan-Dynamin (DG1, DG2), rabbit anti-pan-Endophilin (Nuts), mouse anti-GAD65 (GAD6), rabbit anti-SNAP25 (MC21), rabbit anti-Synapsin (G246), mouse anti-VAMP2 (c69.1), rabbit anti-Synaptophysin (G95) and mouse anti-Syt1 (c41.1).

2) Abcam: rabbit anti-LRRK2 (ab133474, MJFF2 [c41-2])
3) BD Biosciences: mouse anti-α-synuclein (610786, c42), mouse anti-AP2 (611350, AP50) and mouse anti-Hip1R (612118, c44)
4) Cell Signaling Technology: rabbit anti-DARPP-32 (2306, 19A3)
5) Life Technologies: mouse anti-α-adaptin (MA1-064, AP6)
6) Merck Millipore: mouse anti-Amph2 (05-449, 99D), goat anti-ChAT (AB144P), mouse anti-CLC (AB9884), rat anti-DAT (MAB369, DAT-Nt), mouse anti-SV2C (MABN367, 4C8.1) and rabbit anti-TH (AB152)
7) Santa Cruz Biotechnology: mouse anti-β-actin (sc-47778, C4) and mouse anti-Hsc70 (sc-7298, B-6)
8) Sigma-Aldrich: rabbit anti-Auxilin (HPA031182), rabbit anti-GABA (A2052), rabbit anti-GFAP (ZRB2383, 2E8) and rabbit anti-SJ1 (HPA011916)
9) Synaptic systems: rabbit anti-Amphiphysin 1 (120002), rabbit anti-AADC (369003), rabbit anti-SV2B (119102), rabbit anti-SV2C (119202), rabbit anti-Synaptogyrin 3 (103 302), and rabbit anti-Syt11 (270003)
10) FUJIFILM Wako Chemicals: rabbit anti-Iba1 (019-19741)

Secondary antibodies used were all purchased from commercial sources as stated:
1) Life Technologies: donkey anti-mouse IgG (H+L) Alexa Fluor 594 (A21203), goat anti-mouse IgG (H+L) Alexa Fluor 488 (A11001), goat anti-mouse IgG (H+L) Alexa Fluor 594 (A11032), goat anti-mouse IgG (H+L) Alexa Fluor 647 (A21236), donkey anti-rabbit IgG (H+L) Alexa Fluor 488 (A21206), goat anti-rabbit IgG (H+L) Alexa Fluor 488 (A11034), goat anti-rabbit IgG (H+L) Alexa Fluor 594 (A11037), goat anti-rabbit IgG (H+L) Alexa Fluor 647 (A21244), goat anti-rat IgG (H+L) Alexa Fluor 488 (A11006), goat anti-rat IgG (H+L) Alexa Fluor 594 (A11007) and donkey anti-goat IgG (H+L) Alexa Fluor 488 (A11055)
2) LI-COR Biosciences: IRDye 800CW donkey anti-rabbit IgG (926-32213), IRDye 800CW donkey anti-mouse IgG (926-32212), IRDye 680RD donkey anti-mouse IgG (926-68072) and IRDye 800CW goat anti-rat (926-32219)

Validation

rabbit anti-SJ1, validated using SJ1-KO mice.
rabbit anti-Auxilin, validated using Aux-KO mice.
mouse anti-Amphiphysin 1,validated using amph1-KO mice
rabbit anti-pan-Dynamin, validated using dynamin triple KO cells
rabbit anti-pan-Endophilin, validated using endophilin triple KO mice (PubMed: 22099461)
rabbit anti-LRRK2, RRID: AB_2713963, Manufacturer's website states that this antibody is KO validated.
mouse anti-α-synuclein, RRID: AB_2748880
mouse anti-AP2, RRID: AB_398872
mouse anti-Hip1R, RRID: AB_399489
rabbit anti-DARPP-32, RRID: AB_823479
rabbit anti-NPY, RRID: AB_2716286
mouse anti-α-adaptin, RRID: AB_2258307
mouse anti-Amph2, RRID: AB_309738
goat anti-ChAT, RRID: AB_2079751
mouse anti-CLC, RRID: AB_992745
rat anti-DAT, RRID: AB_2190413
mouse anti-SV2C, RRID: AB_2905667
rabbit anti-TH, RRID: AB_390204
mouse anti-β-actin, RRID: AB_2714189
mouse anti-Hsc70, RRID: AB_627761
rabbit anti-Auxilin, RRID: AB_10611957
rabbit anti-GABA, RRID: AB_477652
rabbit anti-GFAP, RRID: AB_2905668
rabbit anti-SJ1, RRID: AB_1857692
rabbit anti-Amphiphysin 1, RRID: AB_887690
rabbit anti-AADC, RRID: AB_2620131
rabbit anti-SV2B, RRID:AB_887803, Manufacturer's website states that this antibody is validated using SV2B KO mice (PubMed: 19381277)
rabbit anti-SV2C, RRID: AB_887803
rabbit anti-Synaptogyrin 3, RRID: AB_2619752, Manufacturer's website states that this antibody is validated using quadruple KO mice of Synaptophysin 1 and 2 and synaptogyrin 1 and 3 (PubMed: 31090538).
rabbit anti-Syt11, RRID: AB_2619994, Manufacturer's website states that this antibody is KD validated using primary microglia (PubMed: 28686317)
rabbit anti-Iba1, RRID: AB_839504
donkey anti-mouse IgG (H+L) Alexa Fluor 594 (A21203), RRID: AB_141633
goat anti-mouse IgG (H+L) Alexa Fluor 488 (A11001), RRID: AB_2534069
goat anti-mouse IgG (H+L) Alexa Fluor 594 (A11032), RRID: AB_2534091
goat anti-mouse IgG (H+L) Alexa Fluor 647 (A21236), RRID: AB_2535805
donkey anti-rabbit IgG (H+L) Alexa Fluor 488 (A21206), RRID: AB_2535792
goat anti-rabbit IgG (H+L) Alexa Fluor 488 (A11034), RRID: AB_2576217
goat anti-rabbit IgG (H+L) Alexa Fluor 594 (A11037), RRID: AB_2534095
goat anti-rabbit IgG (H+L) Alexa Fluor 647 (A21244), RRID: AB_2535812
goat anti-rat IgG (H+L) Alexa Fluor 488 (A11006), RRID: AB_2534074
goat anti-rat IgG (H+L) Alexa Fluor 594 (A11007), RRID: AB_10561522
donkey anti-goat IgG (H+L) Alexa Fluor 488 (A11055), RRID: AB_2534102
IRDye 800CW donkey anti-rabbit IgG (926-32213), RRID: AB_621848
IRDye 800CW donkey anti-mouse IgG (926-32212), RRID: AB_621847
IRDye 680RD donkey anti-mouse IgG (926-68072), RRID: AB_10953628
IRDye 800CW goat anti-rat (926-32219), RRID: AB_1850025

Eukaryotic cell lines

Policy information about cell lines and Sex and Gender in Research

Cell line source(s)	*State the source of each cell line used and the sex of all primary cell lines and cells derived from human participants or vertebrate models.*
Authentication	*Describe the authentication procedures for each cell line used OR declare that none of the cell lines used were authenticated.*
Mycoplasma contamination	*Confirm that all cell lines tested negative for mycoplasma contamination OR describe the results of the testing for mycoplasma contamination OR declare that the cell lines were not tested for mycoplasma contamination.*
Commonly misidentified lines (See ICLAC register)	*Name any commonly misidentified cell lines used in the study and provide a rationale for their use.*

Animals and other research organisms

Policy information about studies involving animals; ARRIVE guidelines recommended for reporting animal research, and Sex and Gender in Research

Laboratory animals	Aux-KO (RRID: MMRRC036980-JAX): Mus musculus, C57BL/6.129/SV, P0 - 12 month old<br>SJ1-KI: Mus musculus, C57BL/6.129S6, P0 - 12 month old.<br>Aux-KO/SJ1-KI: Mus musculus, Cross Aux-KO strain with SJ1-KI, Mus musculus, P0 - 8 month old
Wild animals	The study did not involve wild animals.
Reporting on sex	We did not disaggregate for sex . All data collected were from both sex.
Field-collected samples	The study did not involve samples collected from the field.
Ethics oversight	SingHealth Institutional Animal Care and Use Committee (IACUC)

Note that full information on the approval of the study protocol must also be provided in the manuscript.
